# Pathological and Practical Researches on Diseases of the Brain and Spinal Cord

**Published:** 1833

**Authors:** 


**Art. V.**—*Pathological and Practical Researches on Diseases of the Brain and the Spinal Cord.* By John Abercrombie, M. D., Fellow of the Royal College of Physicians of Edinburgh, &c., and first Physician to his Majesty in Scotland. Philadelphia: Carey & Lea.—pp. 450, 8vo.

In comparing the science of Medicine as it exists at present, with that which has been handed down from the fathers of the profession, we are forcibly struck with the prominent position which pathological anatomy now occupies in its records. Instead of a record of symptoms, faithfully, indeed, and accurately observed, but unintelligible from ignorance of their causes, the scalpel of the anatomist by investigating the derangement of structure upon which they depend has erected a system which, in philosophical accuracy, yields to few of the other sciences. We pretend not to say that it offers a solution to every problem which the ingenuity of man may propose to it. Nature has chosen to invest her ultimate operations with a veil of secrecy; and, in every science, therefore, insurmountable obstacles must eventually obstruct the progress of the bold enquirer. Medicine, too, has its difficulties—difficulties, however, which have been overrated by those cynical objectors who find in the uncertainty of medicine an unfailing source of mirth and ridicule, without reflecting that the same uncertainty pervades every other department of human knowledge.

Among the various forms of disease which have been illustrated by pathological anatomy, there is none, perhaps, in which the improvement is more striking than in the diseases of the brain.

Dr. Abercrombie, the author of this volume, has at different times offered to the profession several works of a pathological and practical nature, which are enriched, not less by the fruits of extensive original observation, than by a judicious and laborious appropriation of the investigations of others. Of these, none is more valuable than the volume now under consideration.

The first part of the work is devoted to the inflammatory affections of the brain. It is well observed by Dr. A. that the investigation of these affections is attended with peculiar difficulties, from the fact that the patient, at an early period, becomes unable to express his feelings, and the proper symptoms of the disease are lost sight of amid that general suspension of the faculties to which we give the name of *oppression of the brain.*

While upon this subject, he further remarks, that the proper course of investigation in diseases of the brain has been lost sight of, and their pathology obscured by the practice of ascribing this oppression to pressure upon the brain. That this opinion is not true is now well ascertained from the fact, that every degree of coma may exist, in connexion with a state of disease that is simply inflammatory without any manifest cause of compression, while, on the other hand, a very considerable degree of effusion may take place without any of the symptoms usually ascribed to compression.

Inflammations of the brain are found to vary very considerably in different cases. "These varieties appear to be referable to three circumstances—the seat of the inflammation—the degree of its activity—and the mode of its termination.

Affections of the brain so frequently occur as idiopathic affections, or supervene upon other diseases, that every medical reader is familiar with the *general symptoms* which indicate inflammation of this organ. We shall not, therefore, notice this part of the work further than to give a general view of these symptoms in the language of the author.

In the Head.—Violent headache with throbbing and giddiness—tinitus—sense of weight and fulness—stupor—a great propensity to sleep. In many obscure and insidious cases, a constant feeling of giddiness is the only remarkable symptom.

In the Eye.—Impatience of light—unusual contraction or dilatation of the pupil—double vision—squinting—blindness—distortion of the eyes outwards—paralysis of the muscles of the eyelids, producing, according to the muscle that is affected, either the shut eye, or the gaping eye—transient attacks of blindness or double vision—objects seen that do not exist—a long.sighted person suddenly recovering ordinary vision.

In the Ear.—Transient attacks of deafness—great noise in the ears—unusual acuteness in the hearing.

In the Speech.—Indistinct or difficult articulation—unusual quickness of speech, or unusual slowness.

In the Pulse.—Slowness and remarkable variations in frequency.

In the Mind.—High delirium—transient fits of incoherence—peculiar confusion of thought, and forgetfulness on particular topics.

In the Muscles.—Paralytic and convulsive affections—sometimes confined to one limb, or even part of a limb ; and a state of rigid contraction of particular limbs.

In the Urine.—There frequently occurs a remarkable diminution of the secretion—sometimes nearly amounting to complete suppression ; and connected with this diminution there is often a frequent desire to pass urine, occasioned probably by the increased acrimony, as the quantity diminishes.—p. 35–6.

It is not necessary to caution a physician of any experience, against the insidious characters of these diseases and the deceitful prospect of amendment which they hold out; since they are matters of every day observation, and the subject of familiar remark. Whenever, therefore, in an attack of fever, symptoms have been observed indicating an unusual affection of the brain, the attentive practitioner will not be led into a fatal security by the apparent subsidence of these symptoms and the return of the pulse to thehealthy standard; still less will his attention remit, if this apparent calm be accompanied with any of the symptoms which have just been enumerated by the author as indications of cerebral disease.

Section II. treats of the *principal seats and terminations* of inflammation of the brain.

The varieties in the seat of the inflammation may be referred to the following heads: 1. The Dura Mater. 2. The Pia Mater and the Arachnoid. 3. The substance of the Hemispheres. 4. The dense white matter forming the central parts of the brain.

As to its terminations, the disease may prove fatal in its

1. Inflammatory stage.

2. By serous effusion.

3. By deposition of false membrane. This arises from inflammation of the membranes. It may be found between the bone and the dura mater or between the dura mater and the arachnoid. Its most common seat is under the arachnoid or on the upper surface of the tentorium.

4. By suppuration. This occasionally takes place in the membranes and sometimes in the ventricles. Its more frequent seat, however, is in the substance of the brain.

5. By ramollisement.

A peculiar disorganization or softening of the brain, which has now received that name,—a term adopted from the French to express the peculiar morbid appearance. It consists in a part of the cerebral substance being broken down into a soft pulpy mass, retaining its natural color, but having lost its cohesion and consistence. It differs entirely from suppuration, having neither the color nor the fetor of pus; but the white parts of the brain in which it is most commonly observed retain their pure milky whiteness. It may be found in any part of the brain; but the most common seat of it in my observations is the dense white matter forming the corpus callosum, fornix and septum lucidum. The septum is generally found in such cases perforated by a ragged irregular opening, and the fornix has either entirely lost both its figure and its consistence, or retains its figure while it is left untouched, but falls down into a soft pulpy mass, when the slightest attempt is made to raise it.—p. 43.

Some difference of opinion has prevailed upon this subject; whether this affection is really the result of inflammation or not; M. Rostan and other French pathologists who have examined the subject with great attention, considering it as a disease *sui generis*. Dr. A. considers it as analagous to gangrene, and attempts to reconcile these conflicting opinions by supposing that, like gangrene in other parts of the body, it may result either from inflammation, or from those derangements of the circulation which are the consequences of age and infirmity.

Gangrene from inflammation is familiar to every one; and equally familiar, though very different in its origin and concomitant symptoms, is gangrene from disease of the arteries of any particular part of the body. Ossification of the arteries of the brain to a very great extent is a common appearance in elderly people, and seems to be a very frequent source of apoplexy with extravasation of blood, at advanced periods of life. It appears extremely probable that it may be the source of that particular condition of a part of the brain which terminates in the ramollissement of M. Roston, and he distinctly points at this explanation of it. On the other hand, I am still disposed to contend, that the ramollissement of young persons occurring in acute affections, and seated chiefly in the central parts, is one of the terminations of inflammation in that particular structure. I conceive it to be an affection of primary importance in the pathology of acute affections of the brain, and to mark a peculiar seat of the inflammation of very frequent occurrence. It is often combined with suppuration in other parts of the brain, and very often with effusion in the ventricles ; but the peculiar interest of it is observed in those cases, in which it is the only morbid appearance, and in which it is sometimes of small extent. Of this some remarkable examples will be given in the sequel, in which the perforation of the septum lucidum, by softening of the fornix, were the only morbid appearances in cases which were fatal with all the usual symptoms of acute hydrocephalus.—p. 45–6.

6. As terminations of the disease in a chronic form we have thickening of the membranes, contraction and obliteration of the sinuses, caries of the bones, &c.

The following remarks—the result of Dr. A.'s observation upon the connexion between the symptoms and the appearances on dissection—are interesting:

In the pathology of acute hydrocephalus, we may consider it as probable, or almost ascertained, that the serous effusion is only one of the terminations of that inflammatory condition of the brain, which is a great and leading object of attention in the pathology and the treatment. Some of the other terminations are scarcely less frequent ; particularly the ramollissement of the central parts, which is sometimes met with as the only morbid appearance, and is found combined with the effusion in a very large proportion of the ordinary cases of hydrocephalus. Other cases, in which the symptoms closely resemble those of hydrocephalus, will be found to terminate by the undefined suppuration, or by this combined with serous effusion, or with the ramollissement of the central parts. In fact, we do not often meet with any one of the terminations uncombined, and it is impossible to anticipate from the symptoms, in what manner the disease may terminate in any particular case. Serous effusion, uncombined with any other morbid appearance, I have usually observed in that which I have described, as the fourth form of the disease, in which the symptoms are slow and insidious in their progress, and at no period exhibit much activity. In the cases of this kind in which the pain is more severe, and the symptoms are more violent, I have generally found either effusion combined with the ramollissement of the central parts, or undefined suppuration. In that which I have described as the second form of the disease,* I have generally observed the encysted abscess or the deposition of false membrane between the arachnoid and pia mater. But these results are by no means uniform ; and the ramollissement in particular may occur with very slight and insidious symptoms. The various terminations, indeed, are very often combined together, and all of them are generally combined with more or less of serous effusion. On what these varieties depend, is at present in a great measure matter of conjecture. There is some reason to believe, that the darker or cortical parts of the brain are the chief seats of suppuration, and that the inflammation of the more central white matter terminates chiefly by

* A form of the disease ushered in by convulsions and followed immediately or speedily by coma or paralysis, and soon terminating fatally.—Ed.

ramollissement. The disease is also greatly modified by the activity of the inflammation, depending probably upon the constitution of the patient. Thus, in some cases, we find it assuming the highest characters of active inflammation, in others, consisting of the pure scrofulous inflammation with the lowest degree of activity, and in others, forming numerous modifications by which these two extreme forms pass one into another by almost insensible gradations.—p. 46–7.

Idiopathic inflammation of the dura mater is a very rare affection, Dr. A. having met with but one distinctly marked case of it. It is more frequently met with as a scrofulous affection, combined with disease of the nose or of the ear. Some habits are extremely liable to severe pain of the ear followed by a discharge of purulent matter. To these cases coma sometimes suddenly supervenes and the patient dies in a few days. On dissection, caries of the pars petrosa of the temporal bone is generally met with, the dura mater in the neighborhood is inflamed, thickened, spongy, and ulcerated, with a deposition either of purulent matter or of false membrane between it and the arachnoid. In some cases a superficial abscess of the brain is found with marks of general disease.

A form of disease similar to this is produced by the extension of disease from the bones of the nose. After a similar train of symptoms, death takes place, and the ethmoid bone or frontal sinuses are found carious, with disease of the membranes and the brain in the neighborhood. The same event may take place from disease of any other part of the bone. Such cases are, of course, generally fatal; instances, however, are recorded in which, on the application of a trephine, they have eventually done well.

As the result of inflammatory action of a more slow and chronic kind, the dura mater is liable to thickening and depositions of new matter betwixt its laminæ.

As inflammation of the arachnoid and pia mater is generally combined, the author does not attempt to distinguish them, but speaks of them under the general term of meningitis. The pia mater frequently presents a most intense degree of inflammation, while the arachnoid seldom exhibits any great degree of vascularity; and hence some have doubted whether the arachnoid ever is the seat of inflammation.

In the symptoms of meningitis there is no uniformity, but the more common mode of its occurrence is by a severe and long continued convulsion sometimes preceded by headache or vomiting; but, in other cases, coming on without any warning. This convulsion passes into coma, which afterwards alternates with a repetition of the convulsion, until, in a few days, the case terminates fatally. In other cases, after the convulsion, the patient appears to be doing well for some time, but afterward falls into coma, either with or without a repetition of the convulsions.

The symptoms are far from uniformly conveying an idea either of the extent or the violence of the inflammation. The whole surface of the brain occasionally exhibiting marks of intense inflammation, when the symptoms have been very obscure, and, *vice versa*, the symptoms being sometimes very severe and well defined, when the inflammation is limited in extent, and runs a protracted course.

The most usual termination is by the formation of a yellow adventitious membrane between the membranes or between them and the adjacent parts. Suppuration may also take place upon the surface of the brain or within the ventricles. In the convulsive affections which occur in children during dentition, a thin but extensive coating of puriform fluid is observed upon the surface of the pia mater.

There is a dangerous modification of this form of disease, which, after death, shews nothing but increased vascularity of the membranes.

It is apt to be mistaken for mania, or, in females, for a modification of hysteria; and in this manner the dangerous nature of it has sometimes been overlooked, until it proved rapidly and unexpectedly fatal. It sometimes commences with depression of spirits, which after a short time passes off very suddenly, and is at once succeeded by an unusual degree of cheerfulness, rapidly followed by maniacal excitement. In other cases, these preliminary stages are less remarkable; the affection, when it first excites attention, being in its more confirmed form. This is in general distinguished by remarkable quickness of manner, rapid incessant talking, and rambling from one subject to another, with obstinate watchfulness, and a small frequent pulse. Sometimes there is hallucination or conception of persons or things which are not present, but in others this is entirely wanting. The progress of the affection is generally rapid; in some cases it passes into convulsion and coma; but in general it is fatal by a sudden sinking of the vital powers, supervening upon the high excitement, without coma. The principal morbid appearance is a highly vascular state of the pia mater, sometimes with very slight effusion betwixt it and the arachnoid. The disease is one of extreme danger, and does not in general admit of very active treatment. General bleeding is not borne well, and the treatment must in general be confined to topical bleeding with purgatives, antimonials, and the powerful application of cold to the head. The affection is most common in females of a delicate irritable habit, but also occurs in males, especially in those who have been addicted to intemperance.—p. 80–1.

Dr. A. in the later years of his practice, has been in the practice of treating these cases by wine and other stimulants. The chief difficulty is in determining the class of cases to which the stimulating treatment is applicable. Whenever "the excitement is accompanied by a small and rapid pulse, and an expression of paleness and exhaustion," he tries this practice, however violent the excitement may be, and, in a considerable number of cases, has found much reason to be satisfied with it.

An affection different in its symptoms, but presenting the same obscurity in its morbid appearances, frequently occurs in connexion with hooping cough and other diseases, generally proving fatal under every mode of treatment.

Chronic disease of the pia mater and arachnoid is met with in various forms, in some cases consisting of thickening of the membranes themselves, in others with depositions of false membrane, and in some with tubercular disease of the pia mater. These appearances are met with in mamiacs and idiots.

*Inflammation of the substance of the Hemispheres.*

In the progress of this disease, considerable modifications occur, arising from the various ways in which the inflammation terminates; in these we are chiefly to attend to the following varieties:

I. *It may be fatal in the inflammatory stage.*

II. *The simple ramollissement.*

III. *The preceding appearance mixed with a proportion of purulent matter.*

IV. *The undefined suppuration.* This might perhaps be considered as a modification of the former, but with the purulent matter predominating in quantity.

V. *The defined or encysted abscess.*

VI. *Ulceration of the surface of the brain.*

We have not room to follow our author through his interesting remarks respecting the various forms of cerebral inflammation and the correspondence which the symptoms bear to the nature of it. "There is every reason to believe," says the author, on a review of the subject, "that this inflammation may exist in various degrees of activity; that in the most active forms it advances speedily to ramollissement or suppuration; but that it also exists in a chronic form in which its progress is very slow, so that it may produce urgent symptoms for a considerable length of time without having advanced beyond that stage in which there is a chance of recovery. This latter form we have reason to believe may afterwards pass into suppuration, or may terminate in induration of the cerebral substance, and thus assume the characters of organic disease. The disease may affect any part of the brain and often appears to commence in a small portion of it, gradually extending over a larger portion." In this manner, probably, we may account for the gradual extension of paralysis from the affection of a single muscle until the disease terminates by fatal coma, or permanent paralysis.

We extract the following remarks upon the pathology of hydrocephalus which he locates in the central parts of the brain—inflammation of the corpus callosum septum lucidum, fornix and the membrane lining the ventricles:

The disease seems to present itself under two different forms. In the one, the inflammation appears to be seated in the membrane lining the ventricles; in the other, in the white matter forming the fornix, septum lucidum, and corpus callosum. In the former case, we find the ventricles filled with a turbid or milky fluid, sometimes containing shreds of coagulable lymph, and sometimes having entirely the characters of purulent matter. These appearances are often combined with a deposition of flocculent matter or false membrane on the surface of the choroid plexus, or on the inner surface of the membrane lining the ventricles, and sometimes with ramollissement of the cerebral matter immediately surrounding them. In the latter case, the affection presents itself in the form of ramollissement or white pulpy degeneration of the parts affected. The septum lucidum is found perforated by a ragged irregular opening, from the softened portion having fallen out. The fornix has lost its consistence in the same manner; and either has lost its figure, by having fallen down into a soft irregular pulpy mass, or, retaining in some degree its figure, while it is not disturbed, falls asunder when the most gentle attempt is made to raise it. The lower part of the corpus callosum is often affected, though, I think, more rarely than the other parts; and there is frequently a similar degeneration of the cerebral matter immediately surrounding the ventricles. It is generally, but not always accompanied by effusion in the ventricles of limpid fluid. The substance which is the product of the disease, is of a pure white color, without fetor, and without the slightest resemblance to purulent matter. It sometimes shows a slightly fibrous texture, but in general is entirely of a soft pulpy consistence without any cohesion of parts.

From all that I have observed of this affection, I have no hesitation in considering it as the termination of inflammation in these particular parts. It is an appearance of very frequent occurrence, and seems to hold an important place in the pathology of the brain, and particularly in the pathology of acute hydrocephalus. A most interesting point in the history of it is, that it may be fatal without effusion, and without any other morbid appearance, though with all the symptoms which are usually considered as indicating acute hydrocephalus.—p. 143–4.

Among the interesting cases given by the author of this affection, we observe some curious anomalies. One in which coma came on suddenly without having been preceded by headache, the pupils dilating upon the approach of light,—another with very obscure symptoms, fatal after the coma disappeared,—a third with remarkable remissions and apparent convalescence, suddenly terminating fatally.

An interesting modification of this affection is that which supervenes upon other chronic diseases, especially of the lungs, the pectoral symptoms generally ceasing after the commencement of the symptoms in the head.

It is the opinion of the author, an opinion which has been also advanced by others, that the prominent symptoms which attend the last stage of hydrocephalus do not arise from effusion, and that we have no reason to believe that mere effusion, if not attended with disease of the central parts of the brain, should necessarily be a fatal disease.

1st. In the ordinary cases of hydrocephalus, the coma and other symptoms attending it are not to be considered as the direct effect of the effusion, but of that morbid condition of the brain of which the effusion is the consequence.

2d. That we have no certain mark which we can rely upon as indicating the presence of effusion in the brain. Slowness of the pulse followed by frequency, squinting, double vision, dilated pupil, paralytic symptoms, and perfect coma, we have seen exist without any effusion.

3d. That all these symptoms may exist in connexion with a state of the brain, which is active, or simply inflammatory, while the disease is the subject of active treatment, and while by such treatment, adopted with decision at an early period, we have the prospect of arresting its progress in a considerable proportion of cases. The ground of prognosis in particular cases depends perhaps in a great measure upon the activity of the symptoms. The more they approach to the character of active inflammation, our prospects of cutting them short will be the greater; and the more they partake of the low scrofulous inflammation, it will be the less. In all of them, the period for active practice is short, the irremediable mischief being probably done at an early period of the disease.—p. 165–6.

*The causes of the inflammatory affections of the brain.* Among the first of the causes enumerated by the author are other febrile diseases, particularly, the exanthemata. One of the most frequent and severe examples of the disease arising from this source is an affection which supervenes upon scarlatina, often during apparent convalescence. If this affection comes on with acute symptoms the patient frequently recovers under active treatment; if it comes on gradually and insidiously, he generally dies. The disease, the author thinks, more frequently arises from *injuries* than the majority of the profession are aware of; the symptoms supervening after so long an interval as to be ascribed to some other cause. *Suppressed evacuations* is another frequent cause, especially suppression of the menses in young women of an unsound constitution. Under this head perhaps may be referred the effusion of the brain, which is apt to supervene upon urinary disorders, particularly Ischuria Renalis.

In this obscure disease, the prominent symptom is a very sudden diminution of the secretion of urine, amounting frequently almost to a complete suspension of it. Generally about the third day from the occurrence of this symptom, the patient is found passing into a state of coma, in which after a few days more he dies. The ventricles of the brain are found distended with fluid.—p. 169.

A full account of this disease with the supervening affection of the brain is contained in Good's Study of Medicine.

The disease often occurs in connexion with chronic or scrofulous disease in other parts of the body; hence it often appears to partake of the chronic form of inflammation which characterises other scrofulous affections.

In such cases the first disease is not properly to be considered as the cause of the head affection. It merely marks the tendency to inflammation of a chronic or scrofulous character; and, in habits so disposed, the affection of the brain may be excited by causes which elude our observation. On the same principle, disease in the brain may appear in connexion with disease of any other organ, especially in unhealthy children. In such cases the liver has often been found diseased; and, founded upon this observation, diseased liver has often been improperly stated as one of the causes of hydrocephalus. The same observation perhaps applies to worms and various other affections of the bowels, which in unhealthy children are often found to accompany hydrocephalus, and have sometimes been considered in the relation of a cause.—p. 170.

It is not necessary to say much upon a subject about which there is so much uniformity of opinion as there is respecting the treatment of inflammation of the brain. The following quotation respecting the efficacy of purging is all that we have at present room for.

In all the forms of the disease, active purging appears to be the remedy from which we find the most satisfactory results; and although blood-letting is never to be neglected in the earlier stages of the disease, my own experience is, that more recoveries from head affections of the most alarming aspect take place under the use of very strong purging, then under any other mode of treatment. In most of these cases indeed, full and repeated bleeding had been previously employed, but without any apparent effect in arresting the symptoms. The most convenient medicine for this purpose is the croton oil.—p. 174.

To the chapter on inflammatory diseases of the brain is added an appendix upon *Tubercular diseases of the Brain*, and *certain affections of the Bones of the Cranium.*

Tubercles in the brain present the same appearances as they do in other parts of the body—they are generally solid bodies of a firm consistence, and whitish color, varying from the size of a pin head to that of a small egg. They are found in every part of the brain, either imbedded in its substance or attached to the membranes; and in the fatal cases they are softened, in a state of partial unhealthy suppuration. They occur in persons or families in whom a tendency to tubercular disease has otherwise manifested itself, and they are often combined with tubercular disease in other organs. The symptoms are of course very obscure, and indeed the disease often proceeds to a very considerable extent without producing any symptoms calculated to excite alarm.

In speaking of the diseases of the bones of the head, Dr. A. mentions an uncommon modification of diseased action in the bones, which is confined to the inner table. The patient had received a severe blow upon the head, by a fall, about a year before. Since that time she had generally complained of fixed pain in the head, with disordered stomach, but without any indication of severe disease of the head, until two or three weeks before her death. On dissection, the external parts of the cranium were found to be natural, but the internal table of the whole of the upper part of the cranium was gone, the dura mater adhering to the rough cancellated structure of the bone by a deposition of soft adventitious membrane of a yellowish color.

Such diseases of the bone are probably the result of a low inflammatory action, originating either from a blow or without any apparent cause. The disease of the internal table alone, is extremely rare. It for the most part affects the external table only, although it sometimes extends to the internal table. Its progress is extremely slow, though when once excited it is impossible to say how far it may extend. It may terminate by exfoliation of the outer table alone, or it may penetrate the whole depth of the bone, and not unfrequently it extends to the dura mater and the brain, and then proves speedily fatal. It often extends slowly and gradually over a very considerable part of the skull, and its progress is so slow, that it sometimes continues to produce unpleasant symptoms through a series of years.

In cases of this kind, Dr. A. thinks there is very little encouragement for the application of the trephine. If matter forms beneath the bone, it should be evacuated, but the only practice that seems justifiable is to make free incisions of the integuments and then promote the separation of the bone by the ordinary means. The uncertainty that must always exist respecting the extent and the exact position of the disease in the bone, and the danger of exposing a surface so liable to inflammation as the membranes of the brain, are the reasons he assigns for this opinion.

The second chapter of this work is devoted to *Apoplectic Diseases*. The author recognizes three distinct forms of this disease, often indeed, running into each other, but still differing remarkably in their symptoms—first, those which are immediately and primarily apoplectic; secondly, those which begin with headache and pass gradually into apoplexy; thirdly, those which are distinguished by palsy and loss of speech without coma. We extract an outline of the symptoms of each form.

I. In the first form of the attack, the patient falls down suddenly, deprived of sense and motion, and lies like a person in a deep sleep; his face generally flushed, his breathing stertorous, his pulse full, and not frequent, sometimes below the natural standard. In some cases convulsion occurs, in others rigid contraction of the muscles of the extremities; and sometimes contraction of the muscles of the one side, with relaxation of the other. In this state of profound stupor, the patient may die after various intervals, from a few minutes to several days; or he may recover perfectly without any bad consequence of the attack remaining; or he may recover from the coma, with paralysis of one side. This paralysis may disappear in a few days, or it may subside gradually, or it may be permanent. Other functions, as the speech, may be affected in the same manner, being speedily or gradually recovered, or permanently lost; and recovery from the apoplectic attack is sometimes accompanied by loss of sight.

II. The second form of the disease begins with a sudden attack of pain in the head; the patient becomes pale, sick, and faint; generally vomits, and frequently, though not always, falls down in a state resembling syncope, the face pale, the body cold, and the pulse very feeble. This is sometimes accompanied by slight convulsion. In other cases, he does not fall down, the sudden attack of pain being only accompanied by slight and transient loss of recollection. In both cases he generally recovers in a few minutes from the first effects of the attack, is quite sensible and able to walk, but continues to complain of headache; after a certain interval, which may vary from a few minutes to several hours, he becomes oppressed, forgetful, and incoherent, and then sinks into coma, from which he never recovers. In some cases paralysis of one side occurs, but in others, and I think the greater proportion of this class, no paralysis is observed.

III. In the third form, the patient is suddenly deprived of the power of one side of the body, and of speech, without stupor; or if the first attack be accompanied by a degree of stupor, this soon disappears; he seems sensible of his situation, and endeavors to express his feelings by signs. In the farther progress of this form of the disease great variety occurs. In some cases, it passes gradually into apoplexy, perhaps after a few hours; in others, under the proper treatment, the patient speedily and entirely recovers. In many cases the recovery is gradual, and it is only at the end of several weeks or months that the complaint is removed. In another variety, the patient recovers so far as to be able to speak indistinctly, and to walk, dragging his leg by a painful effort, and after this makes no further improvement. He may continue in this state for years, and be cut off by a fresh attack, or may die of some other disease without any recurrence of the symptoms in his head. In a fifth variety, the patient neither recovers, nor becomes apoplectic; he is confined to bed, speechless and paralytic, but in possession of his other faculties, and dies gradually exhausted, without apoplexy, several weeks or months after the attack.—p. 222-3.

The first form in a pathological point of view presents three distinct modifications—apoplexy without any morbid symptoms or what the author proposes to call simple apoplexy; apoplexy with serous effusion; apoplexy with extravasation of blood.

Dr. A. contends that there is no ground for the distinction between sanguineous and serous apoplexy, and, secondly, that the apoplectic symptoms do not depend upon compression of the brain, either by extravasated blood, or by the effusion of serum. Leaving the reader to consult the cases from which his inferences are drawn, we give merely the conclusions at which he arrives.

1. There is a modification of apoplexy which is fatal, without leaving any morbid appearance that can be discovered in the brain.

2. There is another modification, in which we find serous effusion, often in small quantity.

3. The cases which are referrible to these two classes are not distinguished from each other, by any such diversity of symptoms as can be supposed to indicate any essential difference in their nature.

4. Without any apoplectic symptoms, we find serous effusion in the brain in an equal or in a greater quantity than in the cases of the second modification.

5. It is therefore probable, that in these cases the effusion was not the cause of the apoplectic symptoms.

6. It is probable, that the cases of the first modification depend upon a cause which is entirely referrible to a derangement of the circulation in the brain distinct from inflammation.

7. It is probable, that the cases of the second modification are, at their commencement, of the same nature with those of the first; and that the serous effusion is to be considered as the result of that peculiar derangement of the circulation, which constitutes the state of simple apoplexy. In other words, it is probable, that the affection which has been called serous apoplexy is to be considered as simple apoplexy terminating by effusion.—p. 235.

The cases not primarily apoplectic, generally arise from a rupture of a blood-vessel, and are almost uniformly fatal. At the commencement of the disease the prominent symptom is a sudden attack of violent headache, with a sudden interruption of the functions of the brain. This is soon recovered from, and the circulation goes on without interruption until a sufficient quantity of blood is effused to produce coma. In other cases the effusion of blood appears to be checked by the formation of a coagulum, and, after a considerable interval, breaks out afresh—in others the effusion takes place in a different part of the brain. The rapidity of the symptoms in these cases will, of course, very much depend upon the size of the ruptured vessel. The coma in some instances rapidly supervenes upon the first attack; in others there is an interval of days, in some instances of one or two weeks.

The rupture generally takes place within the substance of the brain; in some instances, however, the effusion is confined to the ventricles, and Dr. A. gives one instance in which extravasation occurred in all the ventricles and along the whole spinal cord.

The source of the hæmorrhage is various:

1. The most common is the rupture of a vessel of moderate size in the substance of the brain, from which the blood bursts by laceration either into the ventricles or to the surface.

2. The superficial vessels. "This appears," says Dr. A., "to be the meningeal apoplexy of M. Serres."

3. From ulceration and rupture of one of the principal arterial trunks.

4. From the vessels of the choroid plexus.

5. From rupture of one of the sinuses.

6. From rupture of small aneurisms in various parts of the cerebral vessels.

It is the opinion of Dr. A. that the rupture in this form of disease most frequently takes place from disease in the coats of the artery without any relation to that congestive or hemorrhagic condition which seems to constitute the state of simple apoplexy. This disease consists in ossification of the coats; in earthy deposits; and, "in some cases, the inner coat is much thickened, of a soft pulpy consistence and very easily separated." This diseased condition of the arteries may give rise to a variety of complaints in the head, and at length terminate fatally by rupture.

The *Paralytic Cases* comprehend the third class. The leading phenomena in this class is the paralytic affection without coma—in which there is no apoplexy, or the apoplectic state has soon passed off, leaving the paralysis as the more prominent and permanent characteristic.

In the progress of this disease, we observe many remarkable varieties, which may be referred to the following heads:

I. Such an attack may be merely the prelude to the apoplectic, and may pass into it after a short interval. These cases belong chiefly to the second class.

II. The attack may, under the proper treatment, pass off speedily and entirely, leaving, after a very short time, no trace of its existence.

III. The recovery may be very gradual, the use of the affected limbs being restored after several weeks or months.

IV. The palsy may be permanent; that is, the patient, after a certain time, may recover so far as to be able to walk about, dragging his leg with a painful effort, and to speak very imperfectly; and after this, makes no farther improvement to the end of his life, which may be protracted for many years.

V. In a fifth variety the patient makes no recovery; he is confined to bed, speechless and paralytic, but possessed of his other faculties in a considerable degree, and dies gradually exhausted, after several weeks or months; in some cases without coma, in others with coma for a few days before death.—p. 263-4.

The pathology of paralysis is attended with much obscurity. The following, Dr. A. gives as the principal morbid appearances met with, although the disease is frequently observed without any of them, and, on the contrary, they often exist without producing paralytic symptoms:

I. Simple and recent inflammation of the cerebral substance.

II. This inflammation passing into ramollissement.

III. The encysted abscess of the brain.

IV. Induration of a portion of the cerebral substance.

V. Extravasated blood in the ventricles; on the surface of the brain; and in cavities or cysts in the substance of the brain.

VI. The empty cists from which extravasation has been absorbed.

VII. Serous effusion on the surface of the brain.

VIII. Extensive disease of the arteries of the brain.—p. 293.

In addition to these, other causes may be assigned which produce the disease, independent of the brain; viz. long continued exposure to cold; local affections of the nerves; and, "there is a modification which seems to be connected with the state of the circulation in the affected part." Dr. A. gives a case of very extensive paralysis in which the whole arterial system was extensively ossified.

Dr. A. supposes, and there is very little reason to doubt the truth of his opinion, that paralysis may depend upon a deranged condition of the circulation of the brain, which may leave no traces of disease after death. In this manner we are to account for the rapid disappearance of cases of very extensive paralysis, and, what is perhaps still more remarkable, for the recovery of cases that have long continued without any amendment.

When the affection depends upon extravasation, we find a great diversity of symptoms dependent in a very considerable degree upon the condition of the brain. If the surrounding brain be healthy, a very considerable extravasation may take place without producing very severe or permanent symptoms. On the contrary, if a small coagulum produce severe symptoms, it will generally be found combined with some other form of disease in the brain.

When the surrounding cerebral substance is healthy, coagula of considerable size are absorbed. The thinner parts are first taken up, leaving the coagulum of a firm fibrous texture, which also, after a certain time, entirely disappears. As this process is going on the cavity becomes lined with a firm membrane, and when the coagulum has entirely disappeared, we find the cyst remaining and forming a well defined cavity which is generally entirely empty. According to his experience this cavity generally continues until the end of life.

We have already alluded to the opinions of the author upon the subject of ramollissement; viz. that it was a species of gangrene. One form was described as occurring after inflammation—the other he supposed to be produced by disease of the arterial system. Speaking of this last form he uses the following language:

This appears to be a disease of the aged,—the cases described by M. Rostan having chiefly occurred in persons from 70 to 80, and upwards. It is accompanied by symptoms of a paralytic and comatose character, and is frequently complicated with extravasation of blood. I have already alluded to the frequency and the exetnt of the disease of the arterial system of the brain in advanced life; and there appears to be considerable probability in the conjecture, that this may be the source of the ramollissement in the cases of this class. The disease of the arteries consists of ossification, with thickening and contraction, frequently to a great extent, and sometimes with separation of the inner coat. It corresponds precisely with the state of the arteries, which we know to produce gangrene in other parts of the body, particularly in the toes and feet of old people; and, in another place, I have described a remarkable case in which separation of the inner coat of the iliac artery produced gangrene of the whole extremity, which was fatal in four days.—p. 284.

The effect of the disease of the brain upon the nervous system is very diversified. The power of motion is more frequently lost—in some cases the feeling is lost also—in others the feeling is lost without the power of motion being at all affected.

A gentleman who was under the care of Dr. Hay, of Edinburgh, had two paralytic attacks at the distance of eight months from each other. In the first, there was perfect loss of feeling, with only partial loss of motion; in the second there was perfect loss of motion with only partial loss of feeling. He recovered perfectly from the first attack after a short time; but, after the second, though he recovered partially, he continued to drag his leg, and after a year or more died of apoplexy. It is unnecessary to refer the scientific reader to the light which has been thrown on this curious subject by the discoveries of Mr. Charles Bell.—p. 290.

The sensibility of paralytic limbs in some cases is very much increased, and sometimes, they become intensely painful without any apparent cause. As regards the progress, whether of cure or of attack, it sometimes begins at the part of the limb next the body, in other cases it begins at the extremity. Dr. A. has seen a patient who could write legibly, with his arm supported, when the arm from the shoulder to the elbow was completely paralytic.

Dr. A. does not think that the affected limb is generally colder, but that it has lost, in some degree, the power of preserving a medium temperature, and that it partakes of the temperature of the medium to which it is exposed.

Some interesting phenomena are presented by the condition of the mental faculties in paralysis. One of the most common is a loss of the *memory of words*, while the ideas of things and their relations appears to be very little impaired. This has sometimes been observed to be confined to a particular class of words, as nouns, verbs, or adjectives. Sometimes the individual comprehends the word when written, though the relation between the sound and the thing is lost. Another modification consists in placing one name for another, and in using them uniformly in this way. In other cases the patient having forgotten his language, invents arbitrary names by which he makes his attendants sensible of his immediate wants. Individuals also are said to forget one language while they recollect another, and in other cases to retain their recollection of words without associating with them any meaning. A remarkable case was communicated to Dr. A. in which, an individual who had been blind from a paralytic affection for seven years, was found, on suddenly recovering his sight, to have retained his skill in drawing, for which he had been remarkable before the accident. These facts are curious and interesting, but we know too little of the principles upon which they are founded, to apply them to any useful purpose.

In the *Treatment of Apoplexy*, Dr. A. remarks, our remedies are few and simple—"large and repeated blood-letting, active purgatives, cold applications to the head, an elevated position of the body, cool air and the absence of all stimuli," and antimonials, provided, they do do not occasion vomiting in the early stages. This course the author pursues with every class and description of cases, even in those which, from their occurring in old and debilitated persons, have been thought to require a different mode of treatment.

In the extent of our evacuations, indeed, a due regard is certainly to be had to the age and constitution of the patient, and the strength of the pulse; but I think we have sufficient ground for saying, that there are no symptoms which characterize a distinct class of apoplectic affections, requiring any important distinction in the treatment; or in other words, a class, which in their nature do not admit of blood-letting. On this important point, we may refer with some confidence to the facts which have been related. Weakness of the pulse, and paleness of the countenance, we have seen to be frequent symptoms of the worst form of sanguineous apoplexy; and on the other hand we have seen cases terminate by serous effusion, which were accompanied by strong pulse and flushing of the countenance. Finally. we have seen one remarkable case in which there existed every circumstance that could lead us to consider the disease as serous apoplexy, but which was fatal without any effusion; and another in which there was most extensive effusion without any apoplectic symptom. It is likewise to be kept in mind, that in apoplectic affections the strength of the pulse is a very uncertain guide; for nothing is more common than to find it upon the first attack of apoplexy, weak, languid, and compressible, and becoming strong and full after the brain has become in some degree relieved by large blood-letting.—p. 303–4.

The cases of sudden and remarkably recovery which we often see in long standing cases, should teach us to persevere in the use of such remedies as may be employed without impairing the stamina of the constitution. In using these, Dr. A. suggests that the system should be kept low by spare living and occasional evacuations. This, he considers, an essential part of the treatment, and accordingly, he differs entirely from those who hold that the diet, in paralytic cases, ought to be nourishing and restorative.

Dr. A. has advanced the opinion that apoplexy and paralysis may take place without either lesion or compression of the brain. In an appendix to the section on apoplexy he offers some conjectures upon the nature of the derangements of the circulation to which this form of disease is probably to be attributed.

The cranium is a complete sphere of bone, exactly filled by the brain, which is thereby protected from all influence without, except what is communicated through the medium of the blood vessels which enter it. In an organ so situated, the amount of blood circulating in its vessels cannot materially vary; the cavity must be filled; and although the distribution of the fluids may be affected, yet the amount of fluids in the head will be the same. This statement is confirmed by the appearance of the brain in animals bled to death.

While in such cases, all the other organs of the body have been found completely blanched or drained of blood, the brain has in general presented in this respect its usual appearance, and, in some cases, the superficial cerebral veins have even been found in so distended a state, that one writer has proposed the paradox, that animals which have been bled to death, die of apoplexy. The most able and most satisfactory observations on this subject are those of Dr. Kellie, of Leith,* made upon animals bled to death under a variety of circumstances. The brain in most of these cases, presented its usual appearance, its blood-vessels being well filled; while in others, the appearances were still more remarkable. In one, the sinuses were loaded with dark blood, and the vessels of the pia mater were delicately filled with florid blood. In another, the sinuses were loaded with blood, the veins of the pia mater were well filled, and the choroid plexus was remarkably turgid. In a very few only of

*Transactions of the Medico Chirurgical Society of Edidburgh, vol. i.

the examples it is remarked, that the vessels of the brain contained sensibly less red blood than in the other cases, and in all of these there was observed some serous effusion. On the other hand, when these experiments were repeated on other animals after a small opening had been made in the cranium by the trephine, the brain was found as much drained of blood as any other part of the body,—p. 314–5.

Now, if we suppose a due distribution of the blood in the brain to be necessary to the healthful performance of its functions, we have here a cause for derangement of the functions of the brain which does not exist in relation to any other organ. Thus, if causes exist to produce an excess of blood in the arterial system of the brain—if the arterial action be suddenly increased, if the passage of the blood from the arteries to the veins be obstructed, or if the capacity of the venous system be diminished, the balance between these two systems must be destroyed.

On the other hand, if the action of the heart be weakened, and the blood, consequently, be thrown into the head with a diminished impulse, the deficiency in the circulating mass will be made up by accumulations in the venous system. We may thus have symptoms of oppression from either of these opposite conditions of the system.

Of disease of the latter kind we have frequent instances in patients liable to paralytic or apoplectic diseases. By frequent bleedings, low diet, and other antiphlogistic treatment, the countenance becomes pale, the body anasarcous, and the patient is liable to frequent attacks of giddiness and confusion, generally accompanied with palpitation. When these symptoms occur, they are to be considered as an indication that depletion has been carried already too far, and that the health of the patient will be improved by a more generous diet. A similar state of the system we observed in the last stage of diseases of exhaustion, sometimes in grown persons, more frequently in children—the patient lying in a state of insensibility, the pupils dilated, the eyes open and insensible, the face pale, and the pulse feeble. From this state they frequently recover by the use of wine and nourishment. Syncope probably depends upon a similar condition of the vessels; differing from apoplexy only as it depends upon an opposite condition of the system, the symptoms, and the effect upon the sensorial functions, being almost entirely the same.

The third part of this work is devoted to *Organic Diseases* of the brain. By organic diseases are to be understood either permanent changes of the cerebral substance itself, or new formations within the head. They are thus classed by Dr. A.

1. Tumors formed by thickening of the membranes of the brain, or by deposition of new matter between their laminæ.

2. Depositions of a pellucid or semi-pellucid substance having the general properties of albumen, coagulating into a firm mass in the heat of boiling water.

3. A very dense tumor of a uniform white or ash color, exhibiting the appearance and properties of coagulated albumen. This tumor is generally attached to the dura mater, and when upon the external side, they have been known to produce absorption of the bone and arise externally like wens.

4. Tumors resembling those of the former class but, internally, presenting an organized appearance, and a reddish flesh color.

5. Tubercular disease, sometimes firm, sometimes approaching to ulceration.

6. Induration of the cerebral substance.

7. Ossifications. Under this head are to be included both osseous projections from the inner surface of the cranium, and internal ossifications, which are commonly found in the dura mater, most commonly in the falx.

8. Hydatids. This name has been applied to several affections of the brain, some of which do not appear to be really hydatids. Of this kind are the vesicles which are often met with in the choroid plexus; they seem to be merely the loose cellular texture of that organ, elevated into vesicles by a watery effusion; and in a case by Dr. Baillie, they could be injected from the veins. Real hydatids, however, do occur in the brain, as in a case which will be quoted from Zeder, in which there were numerous hydatids, one of them the size of an egg, and containing three small hydatids within it. Cysts containing a watery fluid likewise occur in various parts of the brain; but it is doubtful whether they are to be considered as hydatids.

Respecting the symptoms which accompany these various forms of disease we find no particular symptoms which can be distinctly referred to the various forms of morbid affection. They are referred by Dr. A. to the following heads:

I. The first class is distinguished by long continued and severe headache, without any other remarkable symptom. The pain varies very much both in its seat and in its severity; and one very remarkable character of the affection is, that the pain sometimes occurs in regular paroxysms, leaving intervals of comparative or complete relief. In the more violent paroxysms the pain is intense, obliging the patient to remain for a considerable time in one position, the slightest motion aggravating it to perfect torture; but the remissions from this severe suffering are often so remarkable as to lead a superficial observer into the belief that it is merely periodical headache, or headache connected with dyspepsia. This latter supposition is also countenanced by the stomach being frequently much disordered, and by the more violent attacks being often accompanied by vomiting. The diagnosis, indeed, is sometimes difficult, but, by attention, it will be found that the duration and violence of the pain must lead to a suspicion that the complaint is something more than common headache, and that, though the stomach is at times disordered, yet that the headache is often most severe when no disorder exists in the stomach that can account for it.

II. In the second form, after some continuance of fixed headache, the organs of sense become affected, as the sight, the hearing, the taste and smell, and occasionally the intellect. The loss of sight generally takes place gradually, being first obscured, and after some time lost; and very often one eye is thus affected before the other is at all impaired.

III. The third class corresponds with the second in the pain and affections of the senses, with the addition of paroxysms of convulsion.

IV. The fourth class is distinguished by convulsion, without any affection of the senses, often with very little complaint of pain, and in general without that fixed and constant pain which occurs in the other classes.

V. The fifth class leads our attention to a new set of symptoms, namely, the paralytic. These may occur in the form of hemiplegia, paraplegia, or paralysis of all the parts below the neck, and in some cases one limb only is affected. The disease is distinguished from the ordinary paralytic cases, by coming on more gradually; one limb, perhaps, or part of a limb being first weak, and the weakness extending very gradually, until it amounts to paralysis.

VI. The sixth class calls our attention to a subject of much interest; a train of symptoms, which are referred to the stomach, but which really depend upon disease in the brain. In many of the cases of organic disease of the brain, the stomach is affected; but those to which I now allude, are remarkable from the affection in the stomach being the prominent symptom. In these there is often, through a considerable part of their progress, very little complaint of the head, or no complaint so fixed and urgent as to direct our attention to the brain as the seat of the disease.

VII. The seventh class is distinguished chiefly by slight and transient affections of an apoplectic character, of which I have formerly given some remarkable examples.—p. 331–7.

Upon the treatment of these affections little can be said. They are not to be considered, however, entirely hopeless. As they generally have their origin in inflammatory action there is every reason to believe that their progress may at least be impeded, as well as the condition of the patient rendered more comfortable. The treatment will consist in keeping the system low, by evacutions, spare diet, cold applications to the head, issues or setons in the neck, and avoiding all causes of excitement.

Chapter IV. is devoted to diseases of the spinal cord. The similarity of structure which exists between the córd and the brain must, of course, induce a very considerable analogy between their diseases. The following list given by the author, will shew that an expectation of this kind is not unfounded:

I. Acute inflammation of the membranes, or meningitis of the cord.

II. Inflammation of the body of the cord, terminating by ramollissement or suppuration.

III. Serous effusion in the spinal canal.

IV. Extravasation of blood in the spinal canal, or spinal apoplexy.

V. Fungoid disease and thickening of the membranes.

VI. Induration of the cord.

VII. Compression of the cord by new formations within the canal, as tubercles, albuminous depositions, hydatids, and ossification of the membranes.

VIII. Destruction of a portion of the cord.

IX. Concussion of the spinal cord.

X. Certain affections of the bones of the spine.—p. 343.

The following extracts upon the symptoms and treatment of this class of diseases will conclude our quotations from Dr. A.'s book:

In regard to the treatment of the diseases of the spinal cord, it is not necessary to enter into any long detail, as it must be regulated by the same principles as the corresponding affections of the brain. In the more acute affections, we must, of course, rely chiefly on free general and topical bleeding, assisted by blistering, purgatives, and other usual auxiliaries. When the affection is in a more chronic form, the treatment will consist chiefly in local applications, as topical bleeding, blistering, and issues, aided by the horizontal posture. In the earlier stages of such affections, I think the most satisfactory treatment, after free topical bleeding, is by a succession of blisters, applied first on one side of the spine, and then on the other, in quick succession, and repeated in this manner to a considerable number. In some of the cases great benefit is also obtained from continued moderate purging.

When we review the phenomena which have been observed to accompany the diseases of the spinal cord, we find affections of all the principal organs of the body. In the parts connected with the head and neck, we find distortions of the eyes, convulsive affections of the face, difficulty and loss of speech, loss of voice, contraction of the jaw, resembling trismus, and difficulty of swallowing, which is said, in some cases, to have nearly resembled hydrophobia. In the viscera of the thorax, there have been observed oppression, palpitation, and strong and irregular action of the heart; painful sense of stricture in the region of the diaphragm, and difficulty of breathing, which, in some cases has been permanent, and in others, has occurred in paroxysms, resembling asthma. In the organs of the abdomen and pelvis, we find vomiting, pain of the bowels, resembling colic; tenesmus, involuntary discharge of feces, and retention or incontinence of urine. In the muscular parts we observe convulsions and paralysis; the convulsions in some cases resembling chorea, in others tetanus. We are by no means prepared to say, in the present state of our knowledge, that all these proceed directly from the affections of the spinal cord, especially as we observe remarkable diversities and considerable want of uniformity in the symptoms. But the subject presents to us a field of observation which promises most important and most interesting results. It has also opened up a wide field of conjecture, in regard to the influence of the spinal cord, in several diseases which have hitherto been involved in much obscurity.—p. 403–4–5.

Various diseases have been ascribed to an affection of the spinal cord—spasmodic diseases, epilepsy, colica pictonum, fever, hydrophobia, hysteria, &c. Whatever may be the grounds upon which this location is made in these particular cases, the condition of the spinal cord is well worthy of being enquired into in a great variety of chronic diseases, and the physician will find himself amply rewarded for directing his attention to this subject.

We must here take leave of our author, having already exceeded the limits we had assigned ourselves. It would be superfluous for us to recommend the volume, further than to say it should be in the library of every physician. F.

